# Erysipelas

**Published:** 1828-03

**Authors:** 


### ERYSIPELAS.

### CASE I. *Erysipelas, and Injury of the Head, successfully treated by copious Bleeding.*

ANT. HITCHCOCK, æt. thirty-eight, a soldier, was admitted into ST. GEORGE'S HOSPITAL, November 24th, 1827, with a lacerated wound on the left side of the forehead, which exposed the bone.

He states that four days ago he fell from the top of a coach, was taken up insensible, and conveyed to some hospital, where he remained until the next day; when, having recovered his senses, he left the hospital and went to a public-house; but suffered so excessively from pain in his side and head, that at the end of a couple of days he was brought to St. George's. On admission, there was much pain in the head, with frequent nausea; eyes suffused and heavy, tongue furred, pulse strong and labouring. The bowels had not been opened since the accident, nor had the wound been dressed for three days.

V.S. ad ℥xx.—Cal. gr. ij.; P. Jal. gr. vj. quartis horis.

25th.—The bowels have been opened several times. He passed a quiet night, and the pain of head is relieved; tongue furred; pulse fuller; thirst. The wounds on the head are suppurating and sloughy.

Cat. Lini cap. —V.S. ad ℥x.—Contr Cal. &c.—H. Sal. cum Mag. Sulph. ℥ss. sextis horis.

26th.—Much pain in the head, as well as under the left nipple, where, on examination, it was discovered that one of the ribs was broken. A blush of redness has appeared upon the forehead.

Lotio Spirit. fronti.—Bandage to the chest.—V.S. ad ℥xv.

The erysipelas extended over the forehead, accompanied with pain of head, thirst, furred tongue, and quick hard pulse. He was again bled to twenty ounces; and, on the 28th, the report states that the erysipelas had extended over the right cheek, with much tumefaction of the eyelid. He was dozing and muttering all night, and is very restless this morning. There is less pain of head, and the tongue is cleaner, but the pulse is quick and full, and there is constant nausea. The blood drawn yesterday is cupped, but, instead of the common buffy coat, it is covered with a thick layer of the most perfectly transparent fibrine.

Repet[r] V.S. ad ℥xx.—Cont[r] Med.

29th.—He was bled yesterday to upwards of thirty ounces. The erysipelas is fading, the head is relieved, the febrile disturbance much diminished. From this day, in fact, he got rapidly well, and the erysipelas had entirely disappeared on the 1st December.

The patient lost in all upwards of 100 ounces of blood; and between the 26th, when the erysipelas first appeared, and the 29th, when it began to fade, above seventy ounces were abstracted. We may notice a circumstance which occurred at the last bleeding, and which seems to have turned the balance in the patient's favor. Whilst the blood was flowing from the vein, the erysipelatous blush began to lose its vivid redness; and, before the whole quantity was taken, the patient not being at the time in a state of syncope, the cheek and forehead were as pale as marble. In the course of a few minutes the redness returned, but never so distinctly as before.

Case II. *Erysipelas, with Sloughing of the Cellular Membrane, successfully treated by Incisions.*

A. B., æt. seventy, admitted at St. Bartholomew's, under the care of Mr. Lawrence, with phlegmonous erysipelas of the right leg and thigh. The patient has been always a remarkably strong healthy man till this attack, which commenced about a fortnight previous to his admission by a stiffness and slight pain on the outer side of the foot, followed by shivering, and soon afterwards by swelling and redness. At the time of his admission the swelling extended almost to the groin; the skin was of a deep red colour, exceedingly tense, and the whole limb very painful. He complained of great headache and sickness; he was hot and thirsty, his tongue covered with dark brown fur; bowels open; pulse quick and intermitting.

On Friday morning three incisions were made,—one, two inches long, on the inside of the thigh; another, of about three inches in length, on the upper part of the calf; and a third, smaller than either, just below the last. A great quantity of purulent matter and large sloughs were discharged from the wounds, but there was no great bleeding, and the man declared himself much relieved. The limb was poulticed, and he was ordered an opiate every four hours. In the night, the house surgeon was sent for, as the man was apparently dying. He gave him some brandy, and ordered him

Ammon. Carb. gr. v.; Mist. Camph. ℥jss.; Conf. Aromat. ʒss. secundis horis.—Ext. Hyoscyami gr. v. stat. et rept[r] post horas duas.

By this treatment he revived, and next day was much better. As the incisions seemed hardly sufficient to give vent to the sloughs and matter, and, as the skin was hollow underneath, they were enlarged, and he was ordered

**Quininæ Sulph. gr. ij. ter indies ; et Vin. rubri ℥iv. quotidie.**

After a few days, the appearances were very favorable ; the swelling and redness were very much abated, and the wounds were discharging good pus, and looked healthy. The fibres of the soleus could be seen through the incisions in the calf as clearly as if dissected, and they did not show any disposition to granulate. The general health was improved.

**The limb was ordered to be rolled, and the poultice discontinued.—Repet$^r$ medicamenta.**

The old man has since that time been going on well.

### Case III. *Phlegmonous Erysipelas—Incisions—Fatal Hemorrhage.*

An old man came into St. Bartholomew's Hospital about the beginning of January, on account of an erysipelatous inflammation, which arose from a blow, and which at first had somewhat of an œdematous character, and seemed to get rather better by the application of thirty leeches twice repeated, and a bleeding, accompanied with saline medicine and purgatives. On the 15th of January, he was ordered Haust. Ammon. Subcarb. every sixth hour, which he only took three or four times, as the leg began to inflame and grow painful again, particularly about the foot.

On the 19th, Mr. Lawrence suspected suppuration to have taken place, and an incision, about two inches long, was made across the back of the foot, but, as little bleeding took place at the time, it was encouraged by fomentations and a poultice. It appears, however, that the hemorrhage soon became greater, and the nurse stupidly took no notice of it, but allowed the patient to lose nearly two pints of blood before she sent for the house surgeon, who, after he arrived, had great difficulty in stopping it.

**Ammon. Carb. gr. v.; Mist. Camph. ℥j.; Conf. Aromat. ℈j. ter indies.**

On the 21st, the patient appeared in a very weak state; tongue very foul; his countenance pallid; his eyes sunk; and he could scarcely answer when spoken to; but his pulse was tolerably strong. The leg was much better, the inflammation having subsided, but it was rather œdematous.

24th.—He has somewhat rallied, but still continues very weak. Leg better.

**Decoct. Cinchon. ℥jss. ; Am. C. gr. v. tertiis horis.—Some port wine and brandy.—Tinct. Opii gr. xxx. nocte.**

25th.—Appears rather better. The medicine was changed to

**Quininæ Sulph. gr. ij. ter indies.**

29th.—He gradually sunk, and died last night.

This case, taken along with others of a similar description, shows that the objection to the practice of making free incisions, on the score of danger from hemorrhage, is by no means imaginary. It may, indeed, be said that the fatal result arose from an accidental circumstance, which might have been obviated; but undoubtedly it must always be a very formidable objection to any method of treatment, that it renders the safety of the patient so immediately dependent on the care of the nurse or other unprofessional attendants.

Case IV. *Erysipelas, treated first with Leeches, and afterwards with Stimulants.*

Edward Jackson, a smith, forty years of age, of a bilious complexion, on the 27th of November struck the left hand with a heavy hammer, and lacerated the first phalanx of the middle finger. He went on working till considerable pain and swelling supervened. He applied, on the 29th November, as an out-patient at St. Thomas's Hospital, and was ordered six leeches and a linseed-meal poultice to the fingers. On the 30th November, the swelling extended over the forearm, without any redness of the skin, and he began to complain of chilliness, pain in his bowels, and sickness. He was ordered a dozen leeches to the arm, and to continue the poultice. Notwithstanding the relief which he felt from the leeches, swelling, with considerable redness, appeared on the following day, extending over the forearm, and accompanied with fever.

On the 3d of December, the inflammation had spread over the whole arm, and large vesicles had formed at the back of it. The leeches were repeated.

He was taken into the hospital on the 6th December, under the care of Mr. Travers: twenty-four leeches had been applied the preceding evening. The wound of the middle finger was then in a state of unhealthy suppuration, the hand and arm were not much swelled, and the skin, from the wrist to the shoulder, was of a dark red, deepest about the inside and middle of the arm, and yielding to pressure. There was no hardness, tension, or tenderness to the touch in the affected part, which, after the swelling had been reduced by the leeches, felt quite easy. The patient looked very pale, and complained of want of appetite and rest; his tongue was white; his pulse seventy, very low and compressible.

**He was ordered small doses of Calomel, Tartarised Antimony, and Opium, every six hours; with neutral salts in the morning.**

December 6th.—The redness of the arm, about the elbow, is less; but it is deeper on the shoulder, where the patient complains of darting pain. Delirious during the night; tongue white, no appetite; pulse seventy, very low.

**Ordered Ammon. Carb. gr. v.; Infus. Cascar. ʒx.; Tinct. Humuli ʒss.; Syrup. Aurant. ℥ss. to be taken every three hours.—Repetr Pil. horâ somni.**

8th.—The erysipelas extends over almost the whole back, causing the patient great uneasiness when he lies down. A good night; no appetite; bowels open.

He was ordered four ounces of port-wine daily.

13th.—The erysipelas has extended over the whole of the right hip and buttock. The cuticle is peeling off the back. The wound of the finger is in a state of healthy suppuration.

The cold lotion was discontinued yesterday.

15th.—The erysipelas has not spread any farther, and the cuticle begins to peel off the buttock. Convalescent.

On the above case the reporter makes the following remarks:—We will venture to say, that, if no leeches had been employed, and if, instead of cold applications, warm cloths had been applied to the part, and due attention paid to the state of the bowels in the first instance, the local inflammation would have terminated at the place which it first attacked, without spreading farther. The local inflammation in idiopathic erysipelas is a very harmless disease, as long as it only affects the skin, always terminating in resolution, and not requiring any active local treatment, which can have no other effect than that of repelling the disease to a part where it may prove more injurious. The whole treatment ought to depend on the general state of the health, and be chiefly directed to the deranged state of the bowels.

The question whether erysipelas ought to be treated by incisions or by bark, can only have arisen from the confounding two very different diseases.

The idiopathic certainly never requires incisions, however advantageous they may be in the phlegmonous; and bark has certainly no specific influence over the local complaint, though the constitutional symptoms may require the exhibition of tonics in either species.

---

